# Relationship between tightness of the hip joint and shoulder/elbow injury in high school baseball pitchers: a prospective study

**DOI:** 10.1038/s41598-020-76894-6

**Published:** 2020-11-17

**Authors:** Noritaka Hamano, Hitoshi Shitara, Tsuyoshi Tajika, Tsuyoshi Ichinose, Tsuyoshi Sasaki, Masataka Kamiyama, Ryosuke Miyamoto, Takuro Kuboi, Fumitaka Endo, Atsushi Yamamoto, Kenji Takagishi, Hirotaka Chikuda

**Affiliations:** Department of Orthopaedic Surgery, Gunma University Graduate School of Medicine, 3-39-22 Showa, Maebashi, Gunma 371-8511 Japan

**Keywords:** Medical research, Risk factors

## Abstract

Some studies have reported that upper limb tightness is a risk factor for shoulder/elbow pain in high school baseball pitchers; but there has been insufficient research on the relationship between lower limb tightness and shoulder and elbow pain in pitchers. This study aimed to clarify the correlation among pre-season hip range of motion (ROM) and shoulder and elbow disorders in high school baseball pitchers. We surveyed 125 high school pitchers. Hip ROM was measured in the supine and prone positions. After the season, based on their answers to the self-recorded questionnaire, a “shoulder or elbow injury” was defined as any condition resulting in the pitcher being considered disabled for ≥ 8 days. An independent t-test and logistic regression analysis were used for statistical analysis. Eleven disabled pitchers (9%) were identified during the season. In the injured group, the ROM of the plant side hip with 90° flexed external rotation was smaller than that in the non-injured group. Preseason limited ROM in the plant side hip with 90° flexed external rotation was a risk factor for the occurrence of shoulder/elbow pain in the season.

## Introduction

It has been reported that high school baseball pitchers have more upper limb injuries during the season than fielders in high school^1^. However, studies on shoulder and elbow pain in pitchers have been insufficient. According to medical check-ups in the pre-season, several previous studies have demonstrated risk factors for shoulder and elbow injuries in baseball players^2,3^. Regarding the upper extremities, Shitara et al. reported an imbalance of shoulder internal and external muscular strength as a risk factor for shoulder and elbow pain during the season^4^. In studies on high school baseball and softball players, it has been suggested that shoulder and elbow pain is caused by a decrease in the range of internal rotation of the shoulder and a decrease in the range of horizontal adduction^5^. Thus, tightness is an important problem for shoulder and elbow pain; however, there have been no studies on the relationship between lower limb tightness and shoulder and elbow disorders. Saito et al. found that reduced hip flexion and 90° flexion internal rotation (IR) on both the dominant and non-dominant legs were risk factors for elbow pain in adolescent players^6^. In a study on professional baseball field players, Schiler et al. reported that players with a history of shoulder pain had a smaller ROM of internal rotation of the hip on the non-throwing side than players without a history of shoulder pain^7^. Although several cross-sectional studies have been conducted, none of the studies have shown a positive relationship between shoulder and elbow pain and lower limb tightness. Therefore, it remains unknown whether lower limb tightness is the cause or consequence of shoulder and elbow pain.

The purpose of this study was to prospectively examine the relation between the hip range of motion (ROM) in the preseason and shoulder and elbow pain in the season in high school baseball pitchers.

## Results

### Baseline characteristics

A total of 125 players consented to this study and submitted answers to our questionnaire. Questionnaires were collected from players with a 94% recovery rate. Eleven pitchers had shoulder and elbow pain during the season. There were no significant differences in baseball history between the two groups (8.2 ± 2.0 and 9.1 ± 1.3 years, respectively; n.s.).

### Univariate analysis (Table 1)

**Table 1:** Results of independent t-tests.

Trail leg (deg)	Non-injured (N = 114)		Injured (N = 11)		P value
	Mean	SD	Mean	SD	
Flex	118.1	9.5	119.1	13.5	0.70
IR0	38.9	10.7	37.7	10.8	0.74
ER0	49.3	10.9	50.9	11.3	0.66
IR90	30	11.3	34.5	13.1	0.22
ER90	49.8	12.3	48.8	10.4	0.80
**Plant leg**					
Flex	120.5	9.6	120.1	11.1	0.90
IR0	37	9.4	38.9	8.6	0.54
ER0	50	10.6	47.7	10.8	0.51
IR90	31.7	11.4	35.1	12.5	0.35
ER90	48.3	11.7	40	8.2	0.02*

*P < 0.05.

Within the injured group, the ROM of the plant side ER90 was significantly less than that in the non-injured group (40.0° ± 8.2° and 48.3° ± 11.7°, respectively; P = 0.02).

No statistically significant differences were noted for other comparison items.

After performing a univariate analysis, odds ratios (ORs) were calculated through logistic regression analysis. Based on this, increases of 1° in 90° abducted ER in the plant hip reduced the risk of injuries by an OR of 0.89, respectively (Table 2).

**Table 2:** Result of logistic regression analysis.

	Odd ratio	95% CI	P value
ER90 on the plant side	0.930	0.871–0.993	0.029

CI, confidence interval.

## Discussion

In this study, we showed that in high school baseball pitchers, ER90 on the plant side during the preseason is a risk factor for developing shoulder and elbow pain during the season. This is the first study to identify hip ROM as a risk factor for shoulder and elbow injuries in high school baseball pitchers.

Although the notion that pitching movements are a kinematic chain has often been described in recent years, it has been reported that defects in the chain movement cause subsequent impairment^8–12^. This notion has led to a comprehensive view of the entire chain movement, rather than just the shoulder and elbow as the causes of shoulder and elbow pain. Recent studies have shown that pitchers whose hip ROM was restricted often suffer from injuries to the hip, hamstring, shoulder, and elbow^6,7,13^. Li et al. identified higher rates of hip, groin, and hamstring injuries in players with decreased IR90 on both sides among professional baseball players^13^. Similarly, Saito et al. found that decreased hip flexion and IR90 in both the trail and plant leg were risk factors for elbow pain in adolescent players^6^. In professional baseball players, Scher et al.^7^ reported that the IR of the non-dominant hip in non-pitchers with a history of shoulder injury was significantly decreased compared to that of non-pitchers without a history of shoulder injury; however, no significant difference was observed in the pitchers. In contrast, our current prospective studies have demonstrated that lack of ER90 on the plant side hip is a risk factor for shoulder and elbow pain in high school baseball pitchers. The discrepancy between hip IR in previous studies and ER in the present study might have been caused by differences in the study design and subjects. However, our prospective study has a more relevant study design than the cross-sectional designs of previous studies.

Traditionally, we have categorized pitches into six stages: wind up, stride, arm cocking, acceleration, deceleration, and follow-through^14^. Of these phases, the arm-cocking, acceleration, and deceleration phases subject the shoulder and elbow joints to high degrees of forces and movement.

When the pitcher shifts to the arm-cocking phase, the torso accelerates forward, and at the same time, the hip joint on the dominant arm side extends, abducts, and internally rotates. Conversely, the hip joint on the opposite side bends, abducts, and externally rotates^15^.

Therefore, lack of ER90 at the hip joint on the non-throwing side may result in insufficient transmission of force from the waist to the shoulders and elbows as a subordinate part of the chain movement. This may lead to an increase in the burden on the shoulders and elbows. As the players did not show a significant change in hip ER ROM throughout the season^16^, it was worth investigating whether the dynamics of the range of motion of the hip can trigger disability.

The need for research on injury prevention is growing due to the severity and increasing number of pitching injuries at the competitive level^17^. Considering our findings, interventions by stretching the muscles, such as the gluteus minimus, anterior fibers of the gluteus medius muscle, and tensor fasciae latae throughout the pre-season and throughout the season may prevent in-season disability.

One advantage of this study was that all of the subjects were pitchers; this was because it has been reported that high school baseball pitchers have more upper limb injuries during the season than fielders in high school^1^.

The present study had some limitations. We did not perform imaging tests, including radiography, computed tomography, or magnetic resonance imaging to detect pathological issues in the participants’ hip joints. We did not want them to be unnecessarily exposed to radiation, and we did not have the budget to cover the cost of performing magnetic resonance imaging for every subject because our study was based on a medical check-up.

## Methods

### Participants

Before enrolment in the study, all subjects provided written informed consent. The study was approved by the Institutional Review Board of Gunma University Hospital on January 28, 2015 (Identification number 1003 2015-01-28). All methods were carried out in accordance with relevant guidelines and regulations.

This prospective study surveyed 133 male (age, 15–17 years) high school baseball pitchers in 66 high schools. We performed annual preseason medical check-ups during January and February 2016. The players in this study had no restrictions on pitching.

The following exclusion criteria were applied: (1) could not throw at the time of the medical check-up because of shoulder/elbow pain; (2) any history of hip disorder, such as Perthes disease, slipped capital femoral epiphysis, or any congenital disorder. The questionnaire included dominant hand and baseball-related injuries.

### Medical check-ups

A basic consultation was conducted to determine the players' physical condition and shoulder and elbow conditions before the season. The following physical parameters were assessed: (1) hip ROM (flexion, IR, external rotation [ER]) in the supine position, and (2) hip ROM (IR, ER) in the prone position. The examiners did not know whether the participants had shoulder and elbow pain when performing the hip measurements. All hip ROM data were collected by two orthopedic surgeons using a digital protractor (iGaging, CA, USA). Intra-rater validity and reliability of the goniometer have been previously established in the literature by the same orthopedic surgeons as this study^18^.

### Supine position measurements

#### Flexion

One of the measurers restrained the pelvis of the pitcher from moving, and the other measurer flexed the hip joint with the measurement side knee flexed. The other leg was kept straight on the ground.

#### IR at 90° of hip flexion

One of the measurers restrained the player's pelvis from moving, and the other measurer rotated the hip joint internally and externally with the measurement side knee and hip bent at 90°. The other leg was kept straight on the ground. The angle between the torso and the lower leg was set to IR90 and ER90.

#### Prone position measurements

With one of the measurers holding the athlete's pelvis stationary, the other measurer bent the knee on the measurement side at 90° and rotated the hip joint internally and externally with the flexion at 0°. The other leg was kept straight on the ground. The angle between the perpendicular and the lower leg was set to IR0 and ER0.

### Identification of shoulder and elbow injuries

A questionnaire was distributed to the athletes at the medical examinations. After the end of the season, we evaluated whether the athlete could participate in practice or games due to shoulder and elbow pain. According to previous studies^4^, we defined “a shoulder and elbow injury” as being unable to pitch for more than 8 days due to shoulder and elbow pain. Shoulder and elbow pain due to trauma was excluded.

### Statistical analysis

Pitchers were divided into injured and non-injured groups. The study was performed using an independent *t*-test. Thereafter, logistic regression analysis was performed. We performed all of these analyses using the IBM SPSS Statistics software program (version 22, IBM Japan, Tokyo). A P value < 0.05 was considered significant. When we examined the sample size, we found that at least 70 subjects were needed to detect a statistically significant difference, requiring a power calculation of 80%.

In conclusion, we identified a pre-season decrease in the 90° flexed external rotation on the plant side as a risk factor for shoulder and elbow injuries in high school baseball pitchers.

In the future, the development of prevention programs may help to prevent high school baseball players from developing injuries.

## Data Availability

The datasets generated during and/or analyzed during the current study are not publicly available due to ethics committee disapproval but are available from the corresponding author on reasonable request.
